# Unpredictable Chronic Mild Stress Upregulates Dopamine Receptor Expression Independent of Fatty Acid-Binding Protein 7 Gene Deletion

**DOI:** 10.1007/s11064-026-04753-3

**Published:** 2026-04-20

**Authors:** Huy Lu, Nicole Roeder, Brittany Richardson, John Hamilton, George Lagamjis, Yuji Owada, Yoshiteru Kagawa, Abhisheak Sharma, Panayotis K. Thanos

**Affiliations:** 1 Behavioral Neuropharmacology and Neuroimaging Laboratory on Addictions, Clinical Research Institute on Addictions, Department of Pharmacology and Toxicology, Jacobs School of Medicine and Biomedical Sciences, University at Buffalo, 1021 Main Street, 14203-1016, Buffalo, NY 14068 USA; 2 Department of Psychology, State University at Buffalo, Buffalo, NY USA; 3 Department of Organ Anatomy, Graduate School of Medicine, Tohoku University, Seiryo-cho 2-1, Aobaku, Sendai, 980-8575 Japan; 4 Florey Institute of Neuroscience and Mental Health, University of Melbourne, Parkville, VIC 3052 Australia; 5 Department of Pharmaceutics, University of Florida, Gainesville, FL 32610 USA; 6 Department of Molecular Biology, Adelson School of Medicine, Ariel University, Ariel, Israel; 7 Department of Exercise and Nutrition, University at Buffalo, Buffalo, NY 14203 USA

**Keywords:** Fatty acid-binding protein 7, Unpredictable chronic mild stress, Endocannabinoid, Dopamine, D1 receptors, D2 receptors

## Abstract

Fatty acid-binding protein 7 (FABP7) assists in the intracellular trafficking of endogenous cannabinoids and polyunsaturated fatty acids (PUFAs) and has been implicated for various psychiatric diseases. Rising evidence demonstrates the crosstalk between the endocannabinoid and dopaminergic systems, particularly in response to stress. The present study seeks to examine the role of FABP7 expression under chronic stress conditions and its impact on the dopaminergic system, specifically dopamine D1 receptor (D1R) and dopamine D2 receptor (D2R) levels. Adult male FABP7^+/+^ and FABP7^−/−^ mice underwent 28-day treatment of unpredictable chronic mild stress (UCMS) procedure. After the stress paradigm, D1R and D2R levels were measured with in vitro autoradiography using [^3^H] SCH23390 and [^3^H] Spiperone, respectively. Stressed mice, regardless of genotype, exhibited an increase in D1R binding across the entire striatum (dorsal caudate putamen (CPu), dorsolateral CPu, dorsomedial CPu, ventral CPu, ventrolateral CPu, ventromedial CPu, nucleus accumbens core and shell), substantia nigra and olfactory tract. Additionally, an increase in D2R binding induced by UCMS was observed in the olfactory tract and certain regions of the striatum (dorsal CPu and ventral CPu). The UCMS paradigm upregulates D1R and D2R binding independent of FABP7 gene deletion, suggesting a compensatory role of other FABPs in the brain in maintaining dopaminergic homeostasis. This stress-induced shift in D1R: D2R ratio may underlie the pathogenesis of major depressive disorder and substance use disorder, as well as the high comorbidity among these conditions.

**Supplementary Information:**

The online version contains supplementary material available at 10.1007/s11064-026-04753-3.

## Introduction

The endocannabinoid system (ECS) has been documented for its extensive involvement in various physiological functions, including the stress response. The signaling pathway of ECS is predominantly mediated by the action of cannabinoid receptor type 1 (CB1R) and type 2 (CB2R), with CB1R being highly expressed in the brain, whereas CB2 is more involved in the immune cells [1]. The widely studied endogenous ligands, or endocannabinoids (eCBs), for ECS consist of 2-arachidonylglycerol (2-AG) and anandamide (AEA). While both ligands exert agonistic effects on both cannabinoid receptors, 2-AG acts as a full agonist for CB1R and exists at a higher concentration in the brain compared to AEA, a partial agonist for CB1R [2]. Degradation of AEA is primarily facilitated by fatty acid amide hydrolase (FAAH), whereas 2-AG is degraded by monoacylglycerol lipase (MAGL) [3]. Due to their lipid nature, AEA and 2-AG can readily diffuse across cellular membranes and require intracellular carrier proteins for transport to their respective catabolic enzymes. Fatty acid-binding proteins (FABPs) play a significant role in intracellular fatty acid shuttling, which include eCBs and long-chain polyunsaturated fatty acids (PUFAs) [4]. Distribution of different FABP isoforms vary throughout the body; FABP3, 5, and 7 are the primary lipid shuttlers in the brain [4]. FABP7 is localized in the astrocytes, neural stem cells, and radial glia-like cells in the spinal cord [5, 6]. Pharmacological inhibition of both FABP5/7 increases AEA levels in the brain and promotes analgesia [7]. FABP7 has also been noted for its influence on gene transcription through its transport of docosahexaenoic acid (DHA), which activates peroxisome proliferator-activated receptor (PPAR) [8]. Therefore, FABP7 represents as a promising pharmacological target for ECS modulation, with implications for various psychiatric disorders [9].

The dopaminergic (DAergic) system has been well-documented for its involvement in cognition, motivation, reward, learning, and mood [10]. The DAergic system is mediated by activating specific G-protein coupled receptors (GPCRs), which are categorized into two main families: D1-like family (D1R​ and D5R​), which are Gαs-coupled proteins that promote an excitatory pathway, and the D2-like family (D2R​, D3​R, and D4R​), which are Gαi/o-coupled protein that carry an inhibitory effect [11]. D1 receptor (D1R) can be found mostly on the post-synaptic terminal [12]. Pharmacological stimulation of D1R has been shown to exacerbate drug seeking behavior while antagonizing D1R suppress this behavior [13, 14]. In contrast, D2Rs are autoreceptors that are located on pre- and post-synaptic, which are responsible for modulating neuronal excitability and attenuating DA output [15]. Previous findings have shown that decreased D2R levels have been linked to increased impulsivity and addictive behavior [15–17].

Growing evidence supports the intricate relationship between the ECS and the dopaminergic system. CB1R, a Gαi/o-coupled protein, is co-located in similar brain regions that contain a high population of DA neurons, such as the striatum, NAc, prefrontal cortex (PFC), and ventral tegmental area (VTA) [18, 19]. Activation of CB1R on GABAergic and glutamatergic neurons, found at higher levels among GABAergic neurons [20], inhibits calcium channel activity, thereby decreasing the release of synaptic vesicles and synaptic transmission of these neurons [21–23]. Thus, activation of CB1R indirectly modulating DAergic release, influencing reward-seeking behavior and fear response [24–26]. There is also emerging evidence of hetero-dimeric formation of CB1R and dopamine D2R, which is a dynamic process that allows for the fine-tuning of the DAergic system through dual activation of both receptors [27–29]. Current research suggests the possibility that modifications to the ECS can lead to disturbances in the DAergic system, which can be implicated for various psychiatric diseases.

The ability to effectively manage stress is a significant factor in the development of certain psychiatric diseases such as major depressive disorder (MDD). Chronic stress has been documented to induce changes in both ECS and DAergic systems. While the ECS initially acts as a stress buffer by preventing hyperactivation of the hypothalamic–pituitary–adrenal (HPA) axis, chronic stress can impair this retrograde regulatory mechanism due to the desensitization of CB1R [30]. Therefore, this marks the ECS as a promising pharmacological target and etiological marker for MDD. Furthermore, chronic stress has also been recorded to induce complex changes to the DAergic system. Mild to moderate stressors have an activating effect on DA release, implicating it in addiction-like behaviors; however, chronic unpredictable stressors tend to have an inhibitory effect on DA release, implicating an anhedonia phenotype [31]. However, previous literature has yet to find a unanimous finding on the effect of stress on the expression of DA receptors. Therefore, the present study seeks to navigate the direction of change of stress on DA receptor expression using autoradiographic binding.

Deletion of FABP5 and FABP7 has been previously shown to reduce the anhedonia phenotype, a key feature for MDD [32]. Furthermore, deletion of FABP7 and FABP5 have shown to attenuate stress-induced reinstatement for cocaine place preference, while lowering stress-induced corticosterone levels [33]. Although the precise mechanisms remain unclear, previous findings demonstrated that genetic deletion of FABP7 leads to differential brain glucose utilization and a reduction in CB1R accompanied by an increase in N-methyl-d-aspartate (NMDA) receptors in the ventral striatum [34–37]. This suggests that FABPs play a critical role in the neurobiological mechanisms underlying both stress response and vulnerability to substance use disorders. Given these findings, the present study seeks to also examine the role of FABP7 under chronic stress and its impact on DAergic system, specifically D1R and D2R levels in the brain using in vitro autoradiography.

## Experimental Procedure

### Animals

Male C57BL/6J (FABP7^+/+^) (*n* = 9–10 for each treatment group; mean weight = 25.7 g) and FABP7^−/−^ knockout mice (*n* = 10 for each treatment group; mean weight = 20.9 g) (graciously provided by Drs. Owada and Kagawa and generated as previously described [38]) were used for the experiment. All mice were single-housed in a standard plastic cage (19 × 29 × 12 cm) in a temperature-controlled environment (22 °C) on a reverse light cycle (10:00–22:00). The protocol was approved by the Institutional Animal Care and Use Committee at the State University of New York at Buffalo and the National Institutes of Health Guidelines for the Care and Use of Laboratory Animals.

### Unpredictable Chronic Mild Stress Procedure and Timeframe

**Fig. 1:**
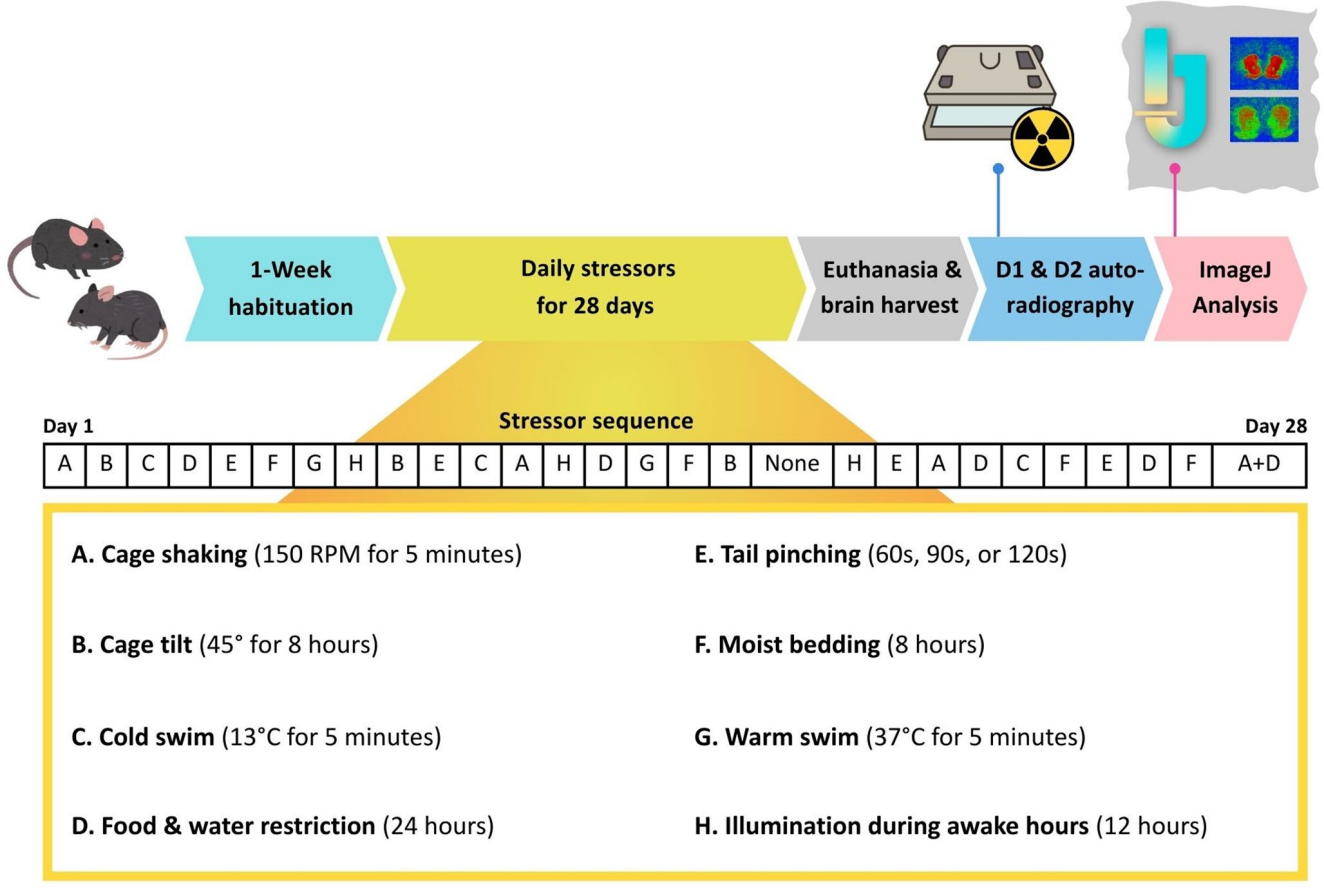
A schematic diagram of experimental design. Different stressors were administered daily to FABP7^+/+^ and FABP7^−/−^ groups throughout the 28-days unpredictable chronic mild stress paradigm

Mice were first habituated to the facility for 1 week prior to the experiment. The UCMS paradigm and related techniques were performed as previously described [34, 35]. FABP7^+/+^ and FABP7^−/−^ mice were randomly divided into two groups (*n* = 9–10/treatment group), with one group undergoing the UCMS procedure while the control group remained in their home cage. The stressed mice were exposed to a variety of stressors over 28 days (Fig. 1). All stress procedures (besides food deprivation, cage tilt, and moist bedding) were carried out in a designated room different from the holding room. After every stressor, mouse health was closely monitored for any abnormality (appearance, behavior, and activity). Following the UCMS procedure, all mice were euthanized using deep isoflurane anesthesia (3%), and brains were rapidly extracted and flash-frozen in 2-isopentane. Brains were then sectioned coronally at 14 μm thick using a Leica cryostat and stored at − 80 °C until receptor bindings were performed.

### D1 Receptor Autoradiography

D1 receptor expressions were determined using [³H] SCH23390 autoradiography and were performed as previously described [39–43]. All sectioned tissues were preincubated for 60 min at room temperature in Tris-HCl buffer (50 nM Tris HCl, 120 mM NaCl, 5 mM KCl, 2 mM CaCl_2_, 1 mM MgCl_2_; pH = 7.4). Slides were air-dried and incubated for 60 min at room temperature in Tris-HCl buffer containing 1.59 nM [³H] SCH23390 (specific activity = 83.2 Ci/mmol, PerkinElmer, USA). Non-specific binding was determined using the adjacent sections and incubated in an identical radioactive buffer with the presence of 1 µM flupenthixol (F114, Sigma Aldrich). Following incubation, 2 × 5 min washes of 4 °C assay buffer solution were performed, and a rapid rinse was done in ice-cold distilled water.

### D2 Receptor Autoradiography

D2 receptor expressions were determined using [³H] Spiperone autoradiography and were performed as previously described [39–43]. All sectioned tissues were preincubated for 60 min at room temperature in Tris-HCl buffer (50 nM Tris HCl, 120 mM NaCl, 5 mM KCl, 2 mM CaCl_2_, 1 mM MgCl_2_; pH = 7.4). Slides were air-dried and incubated for 60 min at room temperature in Tris-HCl preincubation buffer with 0.82 nM [³H] Spiperone (specific activity = 16.2 Ci/mmol, PerkinElmer, USA) and 40 nM ketanserin (S006, Sigma Aldrich). Non-specific binding was determined using the adjacent sections and incubated in an identical radioactive buffer with the presence of 10 µM sulpiride (Cat. No. 0895, TOCRIS). Following incubation, 2 × 5 min washes of 4 °C assay buffer solution were performed, and a rapid rinse was done in ice-cold distilled water.

### Regions of Interest Analysis

**Fig. 2:**
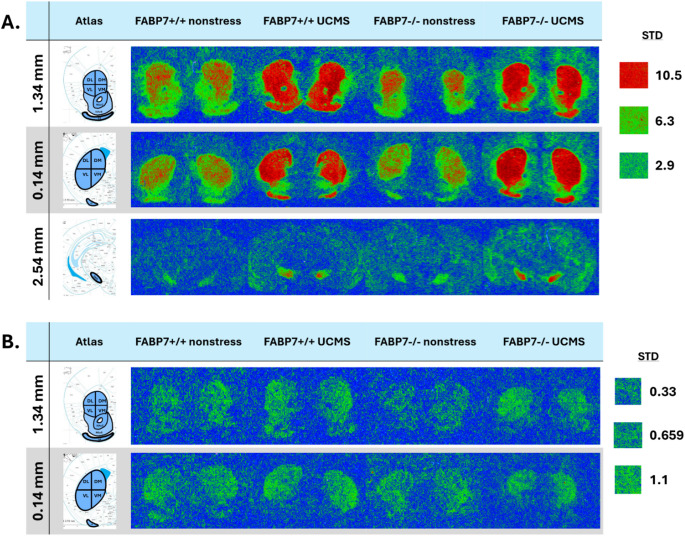
Representative autoradiography images of dopamine receptor binding densities taken at three bregma points (mm). Colorized images from each group and outlined regions of interest are adopted from the Franklin and Paxinos Mouse Brain Atlas, 3rd edition [44]. **(A)** [^3^H] SCH23390 binding; **(B)** [^3^H] Spiperone

Bound slides were subsequently exposed to Biomax XAR film along with a tritium standard for 4 weeks or 8 weeks, retrospective to the ligands. The films were developed, scanned using Brother MFC-J6510DW at 1200 DPI, and images were analyzed using FIJI (ImageJ) software (National Institutes of Health). The regions of interest were taken from each hemisphere following the Franklin and Paxinos mouse brain atlas [44]. The caudate-putamen (CPu) was divided into subregions base on its function: dorsal lateral (DL CPu; habit formation [45]), dorsal medial (DM CPu; goal-directed behavior [46]), dorsal (D CPU; sensory processing and novelty detection [47]), ventral lateral (VL CPu; sensorimotor coordination [48]), ventral medial (VM CPu; motivational, and reward signals [49]), ventral caudal striatum (V CPu; sensory processing and aversive learning [47]); nucleus accumbens core (NAcC), nucleus accumbens shell (NAcS), olfactory tract (OT) and substantia nigra reticular (SNR) (Fig. 2.) Due to low basal level of expression of D2R in the SNR [50], receptor binding was only obtained for D1R.

### Statistical Analysis and Graphing

A two-way ANOVA was conducted within each region of interest to determine the effect of stress (nonstress or UCMS) and genotype (FABP7^+/+^ or FABP7^−/−^) on D1R and D2R binding. The significance level was set at α = 0.05 for each main effect, and post hoc analysis was conducted using Tukey’s multiple comparison test for interaction effects observed. All statistical analyses were performed with Prism8 GraphPad software version 8.4.3.

## Result

### [^3^H] SCH23390 D1 Receptor Binding

**Fig. 3:**
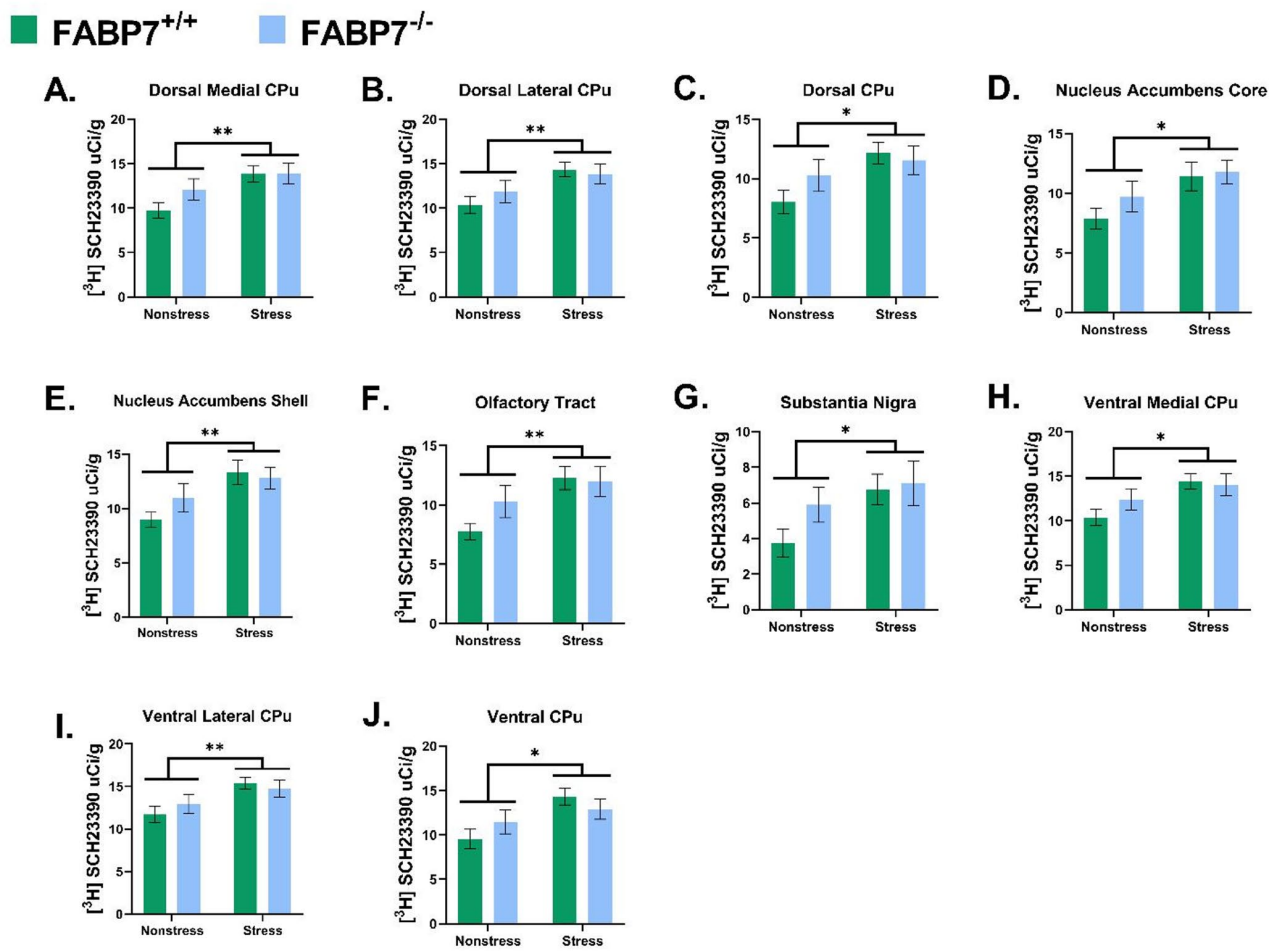
[^3^H] SCH23390 binding within each measured region in FABP7+/+ and FABP7-/- under control versus stress paradigm. **(A)** Dorsal medial caudate putamen (CPu); **(B)** Dorsal lateral CPu; **(C)** Dorsal CPu; **(D)** Nucleus accumbens core; **(E)** Nucleus accumbens shell; (**F**) Olfactory tract; (**G**) Substantia Nigra; **(H)** Ventral medial CPu; **(I)** Ventral lateral CPu; **(J)** Ventral CPu. Two-way ANOVA, *n* = 9–10/group, bar represent mean ± SEM, * indicates statistcally significant difference in an overall stress effect at *p* < 0.05, **p < 0.001

A two-way ANOVA showed a main Treatment effect (nonstress vs. UCMS) on [^3^H] SCH23390 (D1R) binding levels in the DM CPu [F (1,35) = 7.797; *p* = 0.0084, Fig. 3A.], DL CPu [F (1,35) = 8.214; *p* = 0.0070, Fig. 3B.], D CPu [F (1,35) = 5.698; *p* = 0.0225, Fig. 3C.], NAcC [F (1,35) = 0.0163; *p* = 0.0102, Fig. 3D.], NAcS [F (1,35) = 8.413; *p* = 0.0064, Fig. 3E.], OT [F (1,35) = 7.925; *p* = 0.0080, Fig. 3F.], SNR [F (1,35) = 4.670; *p* = 0.0367, Fig. 3G.], VM CPu [F (1,35) = 7.303; *p* = 0.0105, Fig. 3H.], VL CPu [F (1,35) = 8.215; *p* = 0.0070, Fig. 3I.], and V CPu [F (1,35) = 7.383; *p* = 0.0102, Fig. 3J], such that mice who underwent UCMS exhibit an increase in D1R binding compared with the nonstress mice (Table 1.)

**Table 1:** A summary of percent differences in average binding between Nonstress and UCMS mice

ROIs	Dopamine D2R Binding	Dopamine D1R Binding
Dorsal medial CPu	No change	↑ 27.1%
Dorsal lateral CPu	No change	↑ 27.0%
Dorsal CPu	↑ 33.9%	↑ 29.5%
Ventral medial CPu	No change	↑ 25.0%
Ventral lateral CPu	No change	↑ 22.2%
Ventral CPu	↑37.9%	↑ 29.9%
Nucleus accumbens core	No change	↑ 31.8%
Nucleus accumbens shell	No change	↑ 31.0%
Olfactory tract	↑24.8%	↑ 34.2%
Substantia nigra reticular	–	↑ 43.3%

The main effects of Genotype (FABP7^+/+^ vs. FABP7^−/−^) and Genotype–Stress interaction were not found to have a significant effect on [^3^H] SCH23390 binding across the brain regions examined (*p* > 0.05, Table 2 and S1.)

**Table 2:** Result of Two-way ANOVA analysis of [^3^H] SCH23390 binding and [^3^H] Spiperone with the factor of Genotype [FABP7^+/+^, FABP7^−/−^] and Treatment [Nonstress, UCMS] across different regions of interest. Numbers in parentheses indicate the degrees of freedom

Ligand	Regions of Interest	Genotype		Treatment		Interaction	
		F value	p value	F value	p value	F value	p value
[^3^H] SCH23390	Dorsal medial CPu (1, 35)	1.258	0.2696	7.797	0.0084*	1.175	0.2858
	Dorsal lateral CPu (1, 35)	0.2178	0.6436	8.214	0.0070**	0.9014	0.3489
	Dorsal CPu (1, 35)	0.5177	0.4766	5.698	0.0225*	1.595	0.2149
	Ventral medial CPu (1, 35)	0.6384	0.4297	7.303	0.0105*	1.332	0.2563
	Ventral lateral CPu (1, 35)	0.0937	0.7613	8.215	0.0070*	0.9525	0.3358
	Ventral CPu (1, 35)	0.0530	0.8193	7.383	0.0102*	2.114	0.1548
	Nucleus accumbens core (1, 35)	1.042	0.3144	6.365	0.0163*	0.4338	0.5144
	Nucleus accumbens shell (1, 35)	0.5062	0.4815	8.413	0.0064**	1.402	0.2443
	Olfactory tract (1, 35)	1.03	0.3172	7.925	0.0080**	1.666	0.2053
	Substantia nigra reticular (1, 35)	1.667	0.2051	4.67	0.0376*	0.8467	0.3638
[^3^H] Spiperone	Dorsal medial CPu (1, 35)	0.1622	0.6896	0.2731	0.6045	0.4240	0.5192
	Dorsal lateral CPu (1, 35)	0.6437	0.4228	1.931	0.1735	0.2433	0.6249
	Dorsal CPu (1, 35)	0.5234	0.4742	4.299	0.0456*	0.0021	0.9636
	Ventral medial CPu (1, 35)	0.1027	0.7505	0.1908	0.6649	0.2832	0.598
	Ventral lateral CPu (1, 35)	0.0051	0.9437	0.0559	0.8144	0.0183	0.8931
	Ventral CPu (1, 35)	0.1674	0.6849	4.355	0.0443*	0.0015	0.9696
	Nucleus accumbens core (1, 35)	0.0475	0.8287	1.323	0.2579	0.5593	0.4595
	Nucleus accumbens shell (1, 35)	0.6879	0.4125	0.0007	0.9782	4.194	0.0481*
	Olfactory tract (1, 35)	0.4058	0.5282	4.199	0.0480*	3.0861	0.0889

*p < 0.05, **p < 0.001

### [^3^H] Spiperone D2 Receptor Binding

**Fig. 4:**
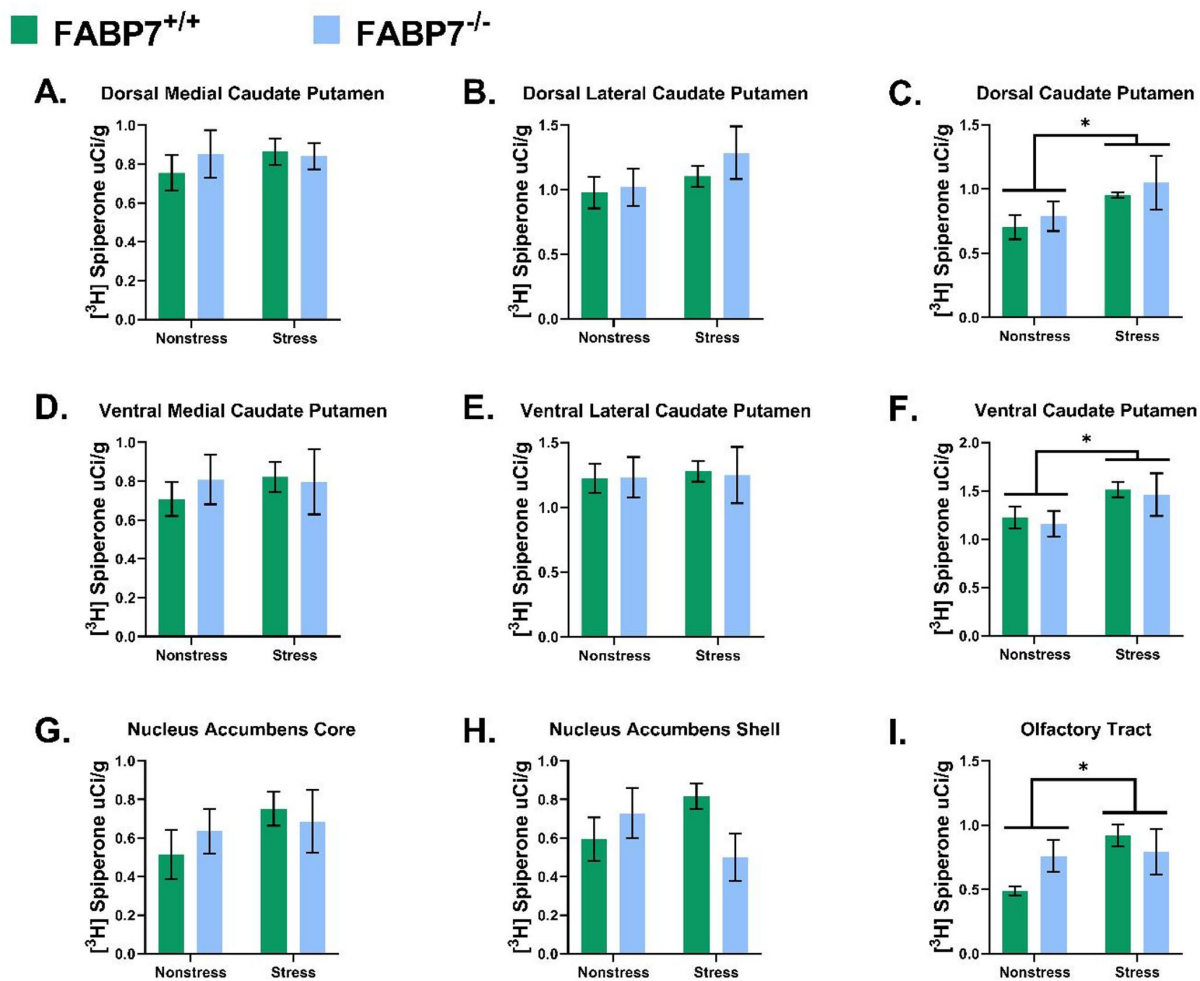
[^3^H] Spiperone binding within each measured region in FABP7^+/+^ and FABP7^−/−^ under control versus stress paradigm. **(A)** dorsal medial caudate putamen (CPu); **(B)** dorsal lateral CPu; **(C)** dorsal CPu; **(D)** ventral medial CPu; **(E)** ventral lateral CPu; **(F)** ventral CPu; **(G)** Nucleus accumbens (NAc) core; **(H)** NAc shell; **(I)** Olfactory tract. Two-way ANOVA, *n* = 9–10/group, bar represent mean ± SEM, * indicates statically significant difference in an overall stress effect at *p* < 0.05

A two-way ANOVA showed a main Treatment effect (nonstress vs. UCMS) on [^3^H] Spiperone binding levels in the D CPu [F (1,35) = 4.299; *p* = 0.0456, Fig. 4C.], V CPu [F (1,35) = 4.355; *p* = 0.0456, Fig. 4F.], and OT [F (1,35) = 7.925; *p* = 0.0080, Fig. 4I.], such that mice who underwent UCMS exhibit an increase in D2R binding compared with nonstress mice.

The main effect of Genotype (FABP7^+/+^ vs. FABP7^−/−^) was not found to have a significant effect on [^3^H] Spiperone binding across the brain regions examined (*p* > 0.05, Table 2.)

 A significant Genotype–Stress interaction was observed in the NAcS [F (1,35) = 4.194; *p* = 0.0481.] However, post hoc analyses using Tukey’s multiple comparison tests did not show any significance across all groups (*p* > 0.05, Table S2.)

## Discussion

Chronic stress exposure has been shown to promote hyperactivity in the hypothalamic–pituitary–adrenal (HPA) axis and disrupt both the ECS and DAergic systems, contributing to anhedonia-like phenotypes and increased vulnerability to mood disorder [31, 51]. Patients diagnosed with MDD frequently present with persistent low mood, anhedonia, and recurrent suicidal ideations [52]. These symptoms are often accompanied by molecular alterations and dysregulation across key biological pathways. To model these depressive-like states in rodents, UCMS paradigms were used to provide insights into the neurobiological mechanisms underlying MDD. Given the intricate role of the FABP7 gene on the ECS [35], and the complexity of the crosstalk between the ECS and DAergic systems indicated above, the present study aims to elucidate the effect of the FABP7 gene deletion on the DAergic system under a chronic stress environment. Intriguingly, we observed that the UCMS paradigm modified the dopaminergic signaling independent of genetic deletion of FABP7. Specifically, UCMS up-regulated D1R binding across the striatum, with an increase ranging from 22.2% in the VL CPu and up to 48% in the SNR (Table 1). However, an increase in D2R binding exerted by UCMS treatment was only observed in selective regions of the striatum—the D CPu (33.9% increase), V CPu (37.9% increase), and OT (24.8% increase).

Previously, Hamilton and colleagues demonstrated that co-deletion of FABP7 and FABP5 increases sucrose consumption and decreases immobility under the forced swim test (FST) [32]. Shimamoto et al. also demonstrated that FABP7^−/−^ alone can reduce immobility of FST, while accompanied by a decrease in time spent in the center area [53]; however, we did not observe any change in the mice’s time spent in the center area based on our previous findings [37]. Additionally, FABP7^−/−^ mice also exhibited anxiolytic-like effects on cognitive processing following traumatic stress [54]. Anti-depressive behaviors can be replicated by other means of CB1R activation. For instance, treatment with other CB1 agonists or AEA-raising compounds have also been shown to reduce immobility time in the FST [55]; whereas, chronic administration of the CB1 blocker rimonabant increases immobility time in the FST [30]. Furthermore, FAAH knockout mice displayed reduced immobility time which was normalized by a CB1 blocker [56].

The complex psychiatric feature of anhedonia is partly attributed to hypodopaminergic tone, which co-aligns with the function of the mesocorticolimbic DAergic system [52]. The system is well-established for controlling cognitive functions, motivated behavior, the central stress response, and the pleasure produced by reinforcers. Given the intricate crosstalk between the ECS and the DAergic systems, CB1 receptor agonists have been shown to enhance DA neuron firing in both the substantia nigra pars compacta and the VTA, as well as increase DA release in the NAc [57]. Specifically, retrograde signaling by the ECS at GABAergic and glutamatergic terminals result in depolarization-induced suppression of inhibitory and excitatory neurotransmission, respectively. While DA neurons in the mid brain region do not express CB1R, the membrane of DA neurons express biosynthetic enzymes for 2-AG at the presynaptic terminal, allowing a retrograde synaptic release of DA [58–60].

Deletion of both FABP5 and FABP7 genes has an anti-anhedonia effect and is presumably mediated by the alteration in the DAergic signaling via a regulatory mechanism described above. We have previously found that FABP5^−/−^ mice display an elevation in D1R in the DM CPu, VM-CPu, and NAc Shell, further alluding to the involvement of FABPs in regulating the dopaminergic system [61]. In the present study, we did not record a significant change in DA receptor binding in FABP7^−/−^ mice. This suggests that FABP5 may play a compensatory role in counteracting the remodeling of DAergic system. One potential factor contributing to the differences between FABP5 and FABP7 is their expression pattern. FABP5 is expressed in both neurons and glia, while FABP7 is most abundantly expressed in astrocytes and more involve in trafficking PUFAs [62]. Previous literature has shown that FABP5^−/−^ mice have an increase in AEA levels in the striatum [63]. Fauzan et al. also demonstrated that FABP5 inhibited phasic 2-AG short-term synaptic plasticity without changing CB1R expression or function while also controlling tonic 2-AG and AEA signaling at striatal GABA synapses of MSN [63]. However, we could not eliminate the possibility of FABP3 playing a similar compensatory role as well. FABP3 is expressed exclusively in the neuron and more phylogenetically closely related to FABP7 compared with FABP5 [64, 65]. FABP3 was detected in D2R-expressing neurons within the rat striatum and was found to be able to bind specifically to the long isoform of D2R in yeast two-hybrid screening and co-immunoprecipitation assays [66]. Although a selective FABP7 inhibitor has not been developed (with SBF-26 currently functioning as a dual FABP5/FABP7 inhibitor), future studies should consider employing selective FABP inhibitors, such as MF1 for FABP3 and ART26.12 for FABP5 [67], to precisely define the role of each FABP subgroup in modulating DAergic system.

UCMS has been shown to exert heterogeneous changes on DAergic system in a region-dependent manner [31]. Our findings on the effects of UCMS on D1R have not previously been reported in previous literature. Prior studies observed selective regional increases in D1R binding [68–70] or no change under UCMS [71–73]. Additionally, the increase in D2R binding recorded in the present study supports Zhang et al.’s work [74], but contrasts with earlier reports that showed stress-induced reductions in the D2R protein and/or transcriptional activity [70, 71, 75]. It is also worth noting that the present study also confounds with our previous findings where UCMS did not affect D1R or D2R binding [73]. A plausible mechanism for this variation could be due to the different types of stressors employed in UCMS paradigms. In the previous study, Delis et al. included paired housing and white noise as part of the UCMS paradigm while excluding food/water deprivation, cold/warm swim, and tail pinching, which were part of the present study [73, 76]. The aim of UCMS is to promote hyperactivity of HPA axis and reduced inhibitory feedback, both of which are key markers for MDD pathophysiology. The modified stressors utilized in the present study (food/water deprivation, cold/warm swim, and tail pinching) have been documented to elicit a heightened HPA response compared to paired housing and white noise [77], suggesting a closer-fitting model for pathophysiology of MDD; however, more investigation is needed to confirm this.

The co-morbidity between MDD and substance use disorders (SUDs) persists at a rate that suggests strong association between the two conditions [78]. In a recent review, De Filippis et al. described the challenges faced in clinical practice establishing the driver in patients with MDD-SUDs (whether MDD leads to more substance consumption or substance consumptions leads to progression in MDD), as well as the inadequate care in patient with MDD-SUDs [79]. Although the etiology of MDD-SUDs remains elusive, the most prevalent hypothesis surrounding the potential predictor behind the disease would be characterized by Reward Deficiency Syndrome (RDS), a work previously coined by Blum’s research team, suggests a hypodopaminergic state and reduction of D2R as the common root of behavioral manifestations [80]. Previous works have shown that chronic stress can lead to a reduction in dopamine in the VTA [31, 81]. Intriguingly, in the present study we also demonstrated that chronic stress leads to imbalance in the D1R: D2R ratio, where there are more D1Rs compared with D2Rs. As depicted in the RDS, a deficiency in D2R also has been recognized as a hallmark for increased drug consumption and implicated for SUDs due to reduction in inhibitory counterbalance signal, the “No-go Pathways” [14, 17, 76, 82]. Contrarily, D1R has been documented to be the driver for drug seeking behavior and reinforcement, stimulating the “Go Pathway” [83–85]. Volkow et al. noted in a review that repeated drug exposure would favor overexpression of the low-affinity D1R and tonic DA response while downregulating high-affinity D2R and phasic DA [14]. Therefore, we speculate that increase in D1R across the striatum in the present study could be implicated in a risk for SUD and sparingly increased in D2R is likely a compensatory response to the increase in D1R across the striatum. More research is needed to test this speculation. These findings, combined with the support of the RDS hypothesis, propose a potential underlying relationship between MDD-SUDs.

The OT has been previously documented for its role in odor-reward and odor-danger associative learning. As part of the ventral striatum, the OT receives significant dopaminergic input from the VTA and is involved in motivated behaviors [86]. D1R expression in the OT has been shown to encode odor valence (whether an odor means reward or punishment) and motivated or avoidance behaviors, depending on the specific OT subdivision [87, 88]; therefore, a stress-induced increase in D1R would be expected to have a similar effect. In contrast, D2R is more selective for odor identity [88], and its activation in the anteromedial OT has been shown to initiate aversive and avoidance behaviors in mice [89]. Our results show that UCMS increases D2R in the OT, suggesting an enhancement in avoidance behavior as typically seen in anhedonic subjects.

## Limitations

The present study acknowledges the existence of limitations. We acknowledge that while the UCMS procedure has been used to promote stress markers (behaviors and cortisol level), we did not evaluate any of the biomarkers. In our prior research, we observed distinct results from genetic deletion of FABPs and pharmacological inhibition of FABPs [32]. It is plausible that FABP7^−/−^ mice differ from wild-type mice in terms of their neural connections, which could make it difficult to characterize the condition. Another limitation of our present study is lack of female data, which restricts our understanding of sex-based differences. Future research should also use pharmacological inhibitors of FABPs to further elucidate the disease’s underlying pathogenesis and conduct additional investigations in female subjects.

## Conclusions

In conclusion, the present study demonstrates that UCMS upregulates D1R and D2R expression in the striatum, regardless of FABP7 gene deletion. D1R expression increased across all analyzed regions (DL CPu, DM CPu, D CPu, VL CPu, VM CPu, V CPu, NAcC, NAcS, OT, and SNR), while D2R upregulation was restricted to select regions (D CPu, V CPu, and OT). Given that UCMS is an established model for depression, these findings suggest that chronic stress may be a key factor in the etiology of MDD-SUDs. Furthermore, the lack of effect of FABP7 deletion on dopamine receptor binding suggests a potential compensatory role by other FABPs, such as FABP3 and FABP5.

## Supplementary Information

Below is the link to the electronic supplementary material.

Supplementary Material 1

Supplementary Material 1

## Data Availability

No datasets were generated or analysed during the current study.
