# Supplementary material for: Unpredictable Chronic Mild Stress Upregulates Dopamine Receptor Expression Independent of Fatty Acid-Binding Protein 7 Gene Deletion
Source: Neurochem Res. 2026 Apr 20;51(3):143. doi: 10.1007/s11064-026-04753-3 (PMC13095973; doi:10.1007/s11064-026-04753-3)
Supplement: Supplementary file 1 — Supplementary Material 1 [file 11064_2026_4753_MOESM1_ESM.docx]

**Supplementary Data**

**Unpredictable chronic mild stress upregulates dopamine receptor expression independent of fatty acid-binding protein 7 gene deletion**

Huy Lu^1^, Nicole Roeder^1,2^, Brittany Richardson^1,2^, John Hamilton^1,2^, George Lagamjis^1,2^, Yuji Owada^3^, Yoshiteru Kagawa^3,4^, Abhisheak Sharma^5^, Panayotis K. Thanos^1,2,6-8*^

*^1^Behavioral Neuropharmacology and Neuroimaging Laboratory on Addictions, Clinical Research Institute on Addictions, Department of Pharmacology and Toxicology, Jacobs School of Medicine and Biomedical Sciences, University at Buffalo, Buffalo 14068, NY, USA.^2^Department of Psychology, State University at Buffalo, Buffalo, NY, USA.^3^Department of Organ Anatomy, Graduate School of Medicine, Tohoku University, Seiryo-cho 2-1, Aobaku, Sendai 980-8575, Japan.^4^Florey Institute of Neuroscience and Mental Health, University of Melbourne, Parkville, VIC 3052, Australia.*

*^5^Department of Pharmaceutics, University of Florida, Gainesville, FL 32610, USA. ^6^Department of Molecular Biology, Adelson School of Medicine, Ariel University, Ariel, Israel. ^7^Department of Orthopedics and Rehabilitation, Stony Brook University, Stony Brook, NY, USA. ^8^Department of Exercise and Nutrition, University at Buffalo, Buffalo, NY, 14203, USA*

**Table of Contents**

1. Post-hoc Tukey’s test results for D1R binding S2

2. Post-hoc Tukey’s test results D2R binding S3

| **Regions of Interest** | FABP7^+/+^ nonstress  vs.  FABP7^-/-^ nonstress | FABP7^+/+^ nonstress  vs.  FABP7^+/+^ UCMS | FABP7^+/+^ nonstress  vs.  FABP7^-/-^ UCMS | FABP7^-/-^ nonstress  vs.  FABP7^+/+^ UCMS | FABP7^-/-^ nonstress  vs.  FABP7^-/-^ UCMS | FABP7^+/+^ UCMS  vs.  FABP7^-/-^ UCMS |
| --- | --- | --- | --- | --- | --- | --- |
| Dorsal medial CPu | 0.4021 | 0.0414* | 0.0462* | 0.6324 | 0.6359 | >0.9999 |
| Dorsal lateral CPu | 0.7417 | 0.0458* | 0.1116 | 0.3289 | 0.5461 | 0.9866 |
| Dorsal CPu | 0.495 | 0.0600 | 0.1524 | 0.6338 | 0.8611 | 0.9811 |
| Ventral medial CPu | 0.5077 | 0.0428* | 0.0874 | 0.5294 | 0.7037 | 0.9946 |
| Ventral lateral CPu | 0.7949 | 0.0438* | 0.1395 | 0.2744 | 0.5574 | 0.9657 |
| Ventral CPu | 0.8002 | 0.0301* | 0.2521 | 0.4065 | 0.9455 | 0.9528 |
| Nucleus accumbens core | 0.8022 | 0.1606 | 0.1055 | 0.8707 | 0.7414 | >0.9999 |
| Nucleus accumbens shell | 0.7023 | 0.0352* | 0.0947 | 0.553 | 0.806 | 0.9997 |
| Olfactory tract | 0.4941 | 0.0339* | 0.0663 | 0.748 | 0.877 | >0.9999 |
| Substantia nigra | 0.5416 | 0.1866 | 0.1218 | 0.9902 | 0.9497 | >0.9999 |

**Table S1.** Result of Tukey’s post hoc analysis of [^3^H] SCH23390 binding across different regions of interest in male FABP7^+/+^ and FABP7^-/-^ under control versus stress paradigm. *p < 0.05

| **Regions of Interest** | FABP7^+/+^ nonstress  vs.  FABP7^-/-^ nonstress | FABP7^+/+^ nonstress  vs.  FABP7^+/+^ UCMS | FABP7^+/+^ nonstress  vs.  FABP7^-/-^ UCMS | FABP7^-/-^ nonstress  vs.  FABP7^+/+^ UCMS | FABP7^-/-^ nonstress  vs.  FABP7^-/-^ UCMS | FABP7^+/+^ UCMS  vs.  FABP7^-/-^ UCMS |
| --- | --- | --- | --- | --- | --- | --- |
| Dorsal medial CPu | 0.8737 | 0.8343 | 0.9164 | 0.9998 | 0.9997 | 0.9981 |
| Dorsal lateral CPu | 0.9961 | 0.9174 | 0.4314 | 0.9745 | 0.5607 | 0.8028 |
| Dorsal CPu | 0.9618 | 0.4758 | 0.2258 | 0.7685 | 0.4608 | 0.9494 |
| Ventral medial CPu | 0.9278 | 0.8984 | 0.9516 | 0.9998 | 0.9999 | 0.9988 |
| Ventral lateral CPu | >0.9999 | 0.9932 | 0.9994 | 0.9961 | 0.9999 | 0.9989 |
| Ventral CPu | 0.9884 | 0.4668 | 0.649 | 0.2954 | 0.4583 | 0.9938 |
| Nucleus accumbens core | 0.8993 | 0.5318 | 0.7758 | 0.9084 | 0.9922 | 0.9825 |
| Nucleus accumbens shell | 0.8185 | 0.4788 | 0.932 | 0.9389 | 0.4789 | 0.2047 |
| Olfactory tract | 0.3334 | 0.0471* | 0.2574 | 0.7434 | 0.9967 | 0.8646 |

**Table S2**. Result of Tukey’s post hoc analysis of [^3^H] Spiperone binding across different regions of interest in male FABP7^+/+^ and FABP7^-/-^ under control versus stress paradigm. *p < 0.05
